# Fetal tubuloglomerular feedback in an ovine model of mild maternal renal disease

**DOI:** 10.14814/phy2.12448

**Published:** 2015-07-14

**Authors:** Anita J Turner, Russell D Brown, Amanda Boyce, Karen J Gibson, A Erik G Persson

**Affiliations:** 1 Australian School of Advanced Medicine, Macquarie UniversitySydney, New South Wales, Australia; 2 Department of Physiology, School of Medical Sciences, University of New South WalesSydney, New South Wales, Australia; 3 Department of Medical Cell Biology, Division of Integrative Physiology, University of UppsalaUppsala, Sweden

**Keywords:** 7NI, micropuncture, nitric oxide, subtotal nephrectomy, TGF

## Abstract

Fetuses of pregnant ewes, which were subtotally nephrectomized prior to mating, were studied to assess whether mild maternal renal impairment would affect fetal tubuloglomerular feedback (TGF) under control conditions and after the inhibition of macula densa-derived nitric oxide (NO). Based on previous observations we hypothesized that, the TGF curve of fetuses of subtotally nephrectomized (STNx) ewes would resemble that of a volume expanded fetus with a high production rate of NO and that inhibition of neuronal nitric oxide synthase (nNOS) would increase the sensitivity of the TGF system in these fetuses. Renal function studies were performed on anaesthetized fetal sheep (133–140 days gestation; term ∼150 days; Isoflurane 2–4% in oxygen). Fetuses were removed from the uterus and placed in a water bath (39.5°C) while maintaining umbilical blood flow. Glomerular filtration rate (GFR) and urine flow rate were markedly increased in fetuses of STNx ewes compared to fetuses of untreated ewes. Interestingly, and contrary to our hypothesis, the fetuses of STNx ewes exhibited no difference in TGF sensitivity in the presence or absence of 7-nitroindazole (7NI; nNOS inhibitor), compared to fetuses of untreated ewes, although sensitivity and reactivity increased in both groups after 7NI. There was however, a decrease in the stop flow pressure and net filtration pressure with an increase in the filtration coefficient (*K*_f_). These factors suggest that maternal renal impairment drives the glomerular hypertrophy which has previously been found to be present in the neonatal period. Thus, we conclude that at ∼138 days gestation, the fetal kidney has matured functionally and fetuses of STNx ewes are able to maintain fluid and electrolyte homeostasis even in the presence of increased transplacental flux.

## Introduction

Maternal renal insufficiency in pregnancy is known to be associated with prematurity, intrauterine growth restriction and risk of fetal loss (Alexopoulos et al. 1996; Trevisan et al. 2004) along with the risk of an accelerated deterioration of the maternal renal disease (Zacur and Mitch 1977; MacCarthy and Pollak 1981; Cunningham et al. 1990; Holley et al. 1996; Jungers et al. 1997). Given that chronic renal disease is often asymptomatic, it is possible that many women become pregnant under conditions of subclinical renal insufficiency; the effect of this on fetal kidney development is the focus of the current study.

Our laboratory has developed an ovine model of chronic maternal renal insufficiency (subtotal nephrectomy, STNx) that produces subclinical signs (Gibson et al. 2006). STNx ewes as well as the fetuses and lambs resulting from timed pregnancies of these STNx ewes, have previously been studied (Gibson et al. 2007, 2008; Brandon et al. 2008, 2011). During late pregnancy, STNx ewes were reported to have unstable sodium balance, although this did not affect fetal growth, blood pressure, heart rate, or arterial blood gases status (Gibson et al. 2006). However, the fetuses did appear to be volume expanded, exhibiting an increased urine flow rate and sodium excretion, and decreased hematocrit, plasma chloride, and circulating renin levels (Gibson et al. 2007). We hypothesized that this reflected an increase in maternal-fetal fluid exchange (Gibson et al. 2007). Interestingly, when lambs born to STNx ewes were studied 1–2 weeks after birth, many of the functional differences did not persist, supporting our hypothesis that the volume expansion had been resolved after birth due to withdrawal from this altered in utero environment (Brandon et al. 2008, 2011).

The TGF mechanism is an essential negative feedback loop which detects alterations in fluid composition at the macula densa in the distal nephron and then regulates afferent arteriolar tone and thus single nephron glomerular filtration rate (SNGFR). When the rate of fluid delivery to the distal nephron increases, this is detected by specialized cells in the macula densa, and TGF causes SNGFR to fall. Conversely, detection of a decreased distal delivery of fluid results in an increase in SNGFR. The TGF system is essential for the maintenance of fluid and electrolyte homeostasis and disturbance of this mechanism has been implicated in the etiology of hypertension (Boberg and Persson 1986; Persson and Boberg 1988). Furthermore, sensitivity of the TGF response is increased in certain rat strains genetically predisposed to hypertension, during the normotensive stage of their development (Dilley and Arendshorst 1984; Persson et al. 1984, 1985).

Nitric oxide (NO) is synthesized in the kidney by neuronal nitric oxide synthase (nNOS) and is important for the maintenance of fluid, electrolyte, and blood pressure homeostasis. nNOS is largely expressed in the macula densa cells of the kidney and has been shown in a number of studies to influence the tubuloglomerular feedback (TGF) mechanism (Mundel et al. 1992; Wilcox et al. 1992; Thorup et al. 1993; Greenberg et al. 1995; Persson et al. 2002). Under normal conditions, NO production via nNOS makes the TGF system less responsive (Wilcox et al. 1992; Thorup and Persson 1996b; Ichihara et al. 1998; Wilcox 1998; Wilcox and Welch 1998).

Volume expansion is known to increase NO production by nNOS (Lahera et al. 1991), reducing the sensitivity and reactivity of the TGF mechanism (Brown et al. 2004), the net effect of which is to promote natriuresis and diuresis and restoration of fluid and electrolyte homeostasis. If the sensitivity is reduced it means that a higher flow is needed to activate the TGF, allowing urine flow rate, and GFR to increase to facilitate fluid loss. It is possible to block this response by inhibiting the production of nNOS (Brown et al. 2004) completely restoring the TGF sensitivity. Dehydration has the opposite effect with the TGF becoming more sensitive (Selen et al. 1983). We have recently shown that the TGF mechanism functions in the fetus in late gestation and that the fetus exhibits a similar response to volume expansion, to the neonatal lamb and adult sheep (Brown et al. 2011).

The aim of the current study was to investigate the role of nNOS on TGF sensitivity in fetal sheep under conditions of mild maternal renal insufficiency. Our hypothesis was firstly that since fetuses of STNx ewes appeared to be volume expanded (Gibson et al. 2007), the TGF curve would resemble that of the volume expanded fetuses studied previously (Brown et al. 2011). Secondly, because of the volume expanded state, inhibition of nNOS would cause a marked increase in TGF sensitivity in the fetuses of STNx ewes and this increase would be more pronounced than in fetuses of untreated ewes.

## Methods

These experiments were approved by the University of New South Wales Animal Care and Ethics Committee.

### Subtotal nephrectomy of nonpregnant ewes

Subtotal nephrectomy was performed on nonpregnant ewes as described previously (Gibson et al. 2006). Briefly, anesthesia was induced via intravenous injection of sodium thiopentone (1 g; Pentothal; Abbott Australasia Pty Ltd, North Ryde, NSW, Australia). The ewe was intubated and anesthesia maintained with 2–4% isoflurane in oxygen (Abbott Australasia Pty, Ltd.).

With the animal positioned on her right side, a paravertebral incision was made under aseptic conditions. The left kidney was located and at least 1 branch of the renal artery was ligated to produce a color change over 30–50% of the renal surface. The wound was closed and the ewe repositioned on her left side. The right kidney was then removed via a second paravertebral incision. The ewes remained under close observation for the following week before being transported back to the university field station. STNx and intact ewes from the same flock were entered into the mating program 2 months after recovery.

### Surgical preparation of fetal sheep

Fetuses were allocated to either undergo blood flow measurements or micropuncture experiments. The initial surgical preparation was identical for both cohorts and has been described previously (Turner et al. 2008). Briefly, anesthesia of ewes was induced (at 133–140 days of gestation) and maintained as described above with the exception that adequate fetal oxygenation was ensured by placing the ewe on a ventilator (Harvard Apparatus model no. 708, South Natick, MA; 16 breaths/min, tidal volume ∼10 mL/kg).

The maternal carotid artery and jugular vein were cannulated and arterial blood pressure and heart rate were measured continuously. Blood gas status was monitored at regular intervals. After exposure of the uterus via a midline abdominal incision, the lower body of the fetus was exteriorized. Both lateral saphenous veins and the left femoral artery as well as the bladder of the fetuses were cannulated for intravenous access, blood pressure and blood gas monitoring, and urine collection, respectively. The ewe was then rolled onto her side, the fetus delivered (maintaining umbilical/placental blood flow) and placed in a temperature regulated shallow water bath on an adjacent table. Fetal temperature was maintained at 39.5°C measured via a rectal thermister. Vecuronium (6 mg to the ewe followed by 2 mg maintenance injections and 0.1 mg/kg to the fetus – Norcuron; Organon Australia, Lane Cove, NSW, Australia) was administered intravenously to the ewe and fetus as needed, to prevent movement.

#### Renal blood flow experiments

A paravertebral incision was made in the left flank of the fetus (Untreated ewe *n* *=* 6, STNx ewe *n* = 8), and Transonic flow probes (Transonic Systems, Ithaca, NY) were placed around both the renal artery and the abdominal aorta proximal to the renal artery (probe sizes 5 mm and 7 mm respectively). Renal artery and abdominal aortic blood flows were measured continuously (Transonic Systems; TS420 transit time perivascular flow meter) and recorded with a Powerlab Chart 5 system (ADInstruments; Castle Hill, NSW, Australia).

#### Micropuncture experiments

In a second cohort of fetuses (Untreated ewe *n* = 8, STNx ewe *n* = 8) a paravertebral incision was made in the left flank of the fetus, the kidney was isolated and placed in a Lucite cup, fixed with a 3% agar solution and bathed in isotonic saline. The outer layers of the renal capsule were removed in order to visualize superficial nephrons using a stereo microscope. Proximal tubular segments on the surface of the kidney were punctured with a sharpened glass pipette (3–5 *μ*m o.d.) filled with 1 mol/L NaCl solution, stained with Lissamine green. Intraluminal injection of Lissamine green was used to visualize the tubular distribution of a single nephron and ensure that early proximal tubular segments were targeted. The pipette was connected to a servo-nulling pressure system (World Precision Instruments, New Haven, CT) to measure proximal tubular free flow pressure (*P*_FF_). To determine stop flow pressure (*P*_SF_), a second pipette (7–9 *μ*m o.d.) was inserted into the same tubule distal to the first, and a wax block placed (with a third pipette) in the tubule between the two pipettes. Tubular pressure upstream to the block was then determined at different loop of Henle perfusion rates (0–40 nL/min). TGF reactivity was assessed by calculating the maximal change in *P*_SF_ (Δ*P*_SF_). TGF sensitivity was assessed by calculating the turning point (TP; the tubular perfusion rate eliciting half–maximal Δ*P*_SF_). Because the magnitude of Δ*P*_SF_ is affected by *P*_SF_ at zero perfusion, we also calculated %Δ*P*_SF_ using the equation %Δ*P*_SF_ = Δ*P*_SF_ / *P*_SF_ × 100 as previously described (Thorup and Persson 1996a). Response curves were obtained by fitting normalized data to the following equation by means of a nonlinear least-squares curve fitting: *P*_SF_ = *P*_SFmin_ + Δ*P*_SF_/1 + *e*^w(PR--TP)^, where *P*_SFmin_ is the average minimum *P*_SF_ when the distal delivery of fluid is increased, PR is the end-proximal perfusion rate and w is the factor determining the width of the perfusion interval during which the *P*_SF_ responded (Selen et al. 1983; Brown et al. 2004).

## Experimental Procedures

Arterial pressure and heart rate of all animals were monitored and recorded continuously using pressure transducers (MLT0670 Disposable BP Transducer; ADInstruments) connected to a Powerlab system and stored for analysis (Powerlab Chart 5, ADInstruments). An intravenous loading dose of lithium chloride was administered to the ewe (150 *μ*mol/kg) and to the fetus (250 *μ*mol/kg). A continuous infusion of lithium chloride in 0.15 mol/L saline at a rate of 10 *μ*mol/kg/h was then commenced for the fetus as well as a maintenance infusion of 0.15 mol/L saline (5 mL/kg/h). There was an equilibration period of 45 min during which fetal urine was drained continuously. Experiments then began (approximately 2.25 h following induction of anesthesia).

Two 30 min control urine collection periods were followed by an intraperitoneal (i.p.) injection of 7-nitro indazole (7NI) (25 mg/kg; Sigma) dissolved in peanut oil (10 mg/mL; Sigma). Collections resumed after 30 min and a further two 30 min urine collections were made. Urine samples were stored at −20°C for later analysis.

### N-nitro-L-arginine-methyl ester (L-NAME) treatment

The TGF mechanism was examined in four untreated and four STNx fetuses after intratubular administration of L-NAME in order to confirm that the effects observed after 7NI treatment were in fact as a result of nNOS blockade in the macula densa. After the initial two 30 min control periods, some fetuses (*n* = 4 in each group) were subject to an infusion of L-NAME (1 mmol/L; Sigma) directly into the tubule via the artificial tubular perfusate (modified Ringer’s solution: 140 mmol/L NaCl, 5 mmol/L KCl, 2 mmol/L CaCl2, 1 mmol/L MgCl2, 4 mmol/L NaHCO3, 7 mmol/L urea, 2 g/L Lissamine green, pH 7.4). TGF was characterized, 7NI administered i.p. and the experiment continued as above.

Fetal and maternal arterial blood samples (7 mL) were taken at the midpoint of the urine collection periods. In addition, arterial blood was collected (0.6 mL) into a heparinized 1 mL syringe for blood gas analysis (ABL715; Radiometer Pacific, Mt. Waverley, Vic., Australia). Hematocrit was measured in duplicate using a microhematocrit centrifuge (Boeco M-24; Hettich, Germany). Remaining blood was centrifuged at 3000 rpm at 4°C for 10 min (Megafuge 1.0 R, Heraeus Sepatech), and plasma stored at −20°C for further analysis.

At the end of the experiments, animals were euthanized (2–5 g pentobarbital sodium, i.v.; Lethobarb, Virbac, NSW, Australia) and fetal body weight and kidney weight were determined.

### Biochemical analysis

Urinary sodium and potassium concentrations were determined by flame photometry (FLM3 Flame Photometer, Radiometer Pacific). Osmolality was measured by freezing point depression (Fiske One-Ten Osmometer; Fiske Associates, Uxbridge, MA). Plasma protein concentrations were measured using a Bradford assay with a BSA standard (Protein Assay Kit II; Bio-Rad Laboratories, Regents Park, NSW, Australia). To determine the plasma protein concentration at the efferent end of the glomerular capillaries, the systemic plasma protein concentration was divided by (1-Filtration fraction) (Andreucci et al. 1971; Navar et al. 1977). For these calculations filtration fraction was expressed as GFR/RPF, and we assumed that filtration fraction was the same in all nephrons (Kallskog et al. 1975). Fetal plasma colloid osmotic pressure (COP_F_) was calculated from the fetal plasma protein concentration (PP_F_) using the equation COP_F_ = 2.24 × PP_F_ − 0.186 (Lumbers et al. 1991). Plasma bicarbonate concentration was calculated using a modification of the Henderson–Hasselbach equation derived by Armentrout et al. (1977).

GFR was determined by endogenous creatinine clearance. Creatinine levels in plasma and urine were determined by the method of Haeckel (1980) and using a microplate reader (model 680 XR; Bio-Rad Laboratories) at 510 nm. Fractional reabsorption of lithium was calculated to provide an index of fractional reabsorption of sodium by the proximal tubule (Thomsen et al. 1981; Lumbers et al. 1988). Plasma and urinary lithium concentrations were measured by atomic absorption spectrophotometry (Varian-Techtron, Melbourne, Australia). Net filtration pressure at the afferent end of the glomerular capillaries (NFP_aff_) was calculated as NFP_aff_ = *P*_SF_ − *P*_FF_ (Andreucci et al. 1971). Net filtration pressure at the efferent end of the glomerular capillaries (NFP_eff_) was estimated by subtracting the rise in COP as a result of glomerular filtration (ΔCOP) from NFP_aff_, that is, NFP_eff_ = NFP_aff_ − ΔCOP. The average net filtration pressure along the glomerular capillaries was estimated as NFP_av_ = (NFP_aff_ + NFP_eff_) / 2 (Andreucci et al. 1971). The total kidney filtration coefficient (*K*_f_) was estimated using total kidney GFR and NFP_av_ (*K*_f_ = GFR / NFP_av_) (Navar et al. 1977).

## Data analysis

Comparisons between Untreated and STNx values in the control period and in the 7NI period were made using a two-way ANOVA for repeated measures. For these comparisons for micropuncture data only, a two-way ANOVA was used without repeated measures, as not all measurements could be obtained in both treatments in all animals. Micropuncture results comparing intratubular L-NAME and systemic 7NI to control values were analyzed using a one-way ANOVA, with a Dunnett’s post hoc test performed to detect differences from control. Statistical analysis was performed using GraphPad Prism version 4.03 for Windows (GraphPad Software, San Diego, CA). All results are expressed as mean ± standard error of the mean (SEM). Differences were considered to be statistically significant if *P* < 0.05.

## Results

### Maternal physiology

STNx ewes were heavier than untreated ewes (65.4 ±1.4 kg, *n* = 16 vs. 56.8 ± 2.3 kg, *n* = 14; *P* < 0.01), and their mean arterial pressure (MAP) was higher (74.9 ± 3.8 mmHg vs. 60.9 ± 2.9 mmHg; *P* < 0.05) but there was no difference in heart rate. Plasma chloride levels of STNx ewes were lower than untreated ewes (103.3 ± 0.6 mmol/L vs. 107.1 ± 0.4 mmol/L; *P* < 0.001). Maternal hematocrits were similar (STNx, 26.9 ± 0.8% vs. 24.9 ± 0.8%, ns) but plasma osmolality was higher in STNx compared to untreated ewes (299 ± 2 mosm/kgH_2_O vs. 293 ± 1 mosm/kgH_2_O, *P* < 0.05). Pco_2_ was also higher in STNx ewes compared to untreated ewes (35.2 ± 1.8 mmHg vs. 28.5 ± 1.3 mmHg, *P* < 0.01), but there was no difference between the groups in arterial Po_2_ or pH.

### Fetal morphology

Fetuses were weighed at the conclusion of the experiment. There was no difference between fetuses of untreated ewes (body weight: 4.8 ± 0.3 kg, kidney weight: 24.9 ± 1.8 g) and fetuses of STNx ewes (body weight: 4.8 ± 0.2 kg, kidney weight: 26.2 ± 1.8 g) with regard to body or kidney weight.

### Untreated versus STNx fetuses

#### Baseline cardiovascular function and blood composition

STNx fetuses exhibited higher arterial Pco_2_ and plasma sodium and bicarbonate concentrations and lower plasma chloride and creatinine levels compared to untreated fetuses (Table1). There was no difference in MAP, heart rate, abdominal aortic (AA) blood flow, renal blood flow (RBF), or renal vascular resistance between untreated and STNx fetuses (Table1).

**Table 1:** Fetal plasma composition, cardiovascular and blood flow parameters

	Control		7NI		*P* value
	Untreated	STNx	Untreated	STNx	
MAP (mmHg)	46.9 ± 1.0	46.8 ± 1.3	43.9 ± 0.9	46.2 ± 1.2	###,†
Heart rate (bpm)	136 ± 5	137 ± 4	127 ± 4	135 ± 6	#
Arterial Po_2_ (mmHg)	25.0 ± 1.5	24.2 ± 1.2	21.5 ± 1.5	21.5 ± 1.2	###
Arterial Pco_2_ (mmHg)	43.1 ± 1.4	53.5 ± 2.8	49.1 ± 2.1	57.5 ± 2.4	**,###
Arterial pH	7.37 ± 0.01	7.33 ± 0.02	7.32 ± 0.02	7.30 ± 0.02	###
Hematocrit (%)	41.4 ± 1.1	43.0 ± 1.7	42.2 ± 1.1	43.8 ± 1.8	###
Sodium (mmol/L)	133 ± 0.5	135 ± 0.4	134 ± 0.7	136 ± 0.5	*,##
Potassium (mmol/L)	4.0 ± 0.1	3.9 ± 0.1	4.1 ± 0.1	3.9 ± 0.1	†
Chloride (mmol/L)	102 ± 1	101 ± 1	102 ± 1	98 ± 3	*
Bicarbonate (mmol/L)	24.0 ± 0.4	26.6 ± 0.5	23.8. ± 0.5	26.7 ± 0.5	***
Glucose (mmol/L)	0.6 ± 0.0	0.6 ± 0.0	0.6 ± 0.1	0.6 ± 0.1	–
Lactate (mmol/L)	4.4 ± 0.3	4.2 ± 0.4	5.1 ± 0.3	4.5 ± 0.4	##
Osmolality (mosm/kg H_2_O)	289 ± 2	293 ± 2	290 ± 1	294 ± 2	–
Creatinine (mg/dL)	1.93 ± 0.18	1.44 ± 0.13	1.94 ± 0.20	1.46 ± 0.14	*
Protein (g/100 mL)	3.84 ± 0.12	4.16 ± 0.08	3.86 ± 0.1	4.03 ± 0.12	–
Colloid osmotic pressure (mmHg)	8.42 ± 0.28	9.13 ± 0.18	8.47 ± 0.22	8.84 ± 0.27	–
Abdominal aortic (AA) blood flow (mL/min/kg)	129 ± 13	159 ± 7	105 ± 17	137 ± 5	###
Total renal artery blood flow (mL/min/g total KW)	3.1 ± 0.3	3.4 ± 0.4	2.8 ± 0.4	3.0 ± 0.4	#
Total renal blood flow (mL/min/kg BW)	16.3 ± 1.7	19.1 ± 2.7	14.8 ± 2.0	17.0 ± 2.3	#
Total renal blood flow (% of AA blood flow)	13.5 ± 2.5	12.4 ± 2.1	16.7 ± 4.5	12.6 ± 2.0	–
Renal vascular resistance (units) MAP(mmHg)/RBF (mL/min/gKW)	15.8 ± 2.8	15.1 ± 2.3	17.0 ± 2.9	17.1 ± 3.1	–

Untreated *n* = 14; STNx *n* = 16. Blood flow fetuses only: Untreated *n* = 5; STNx *n* = 8.

AA, abdominal aorta; KW, kidney weight; BW, body weight; RBF, renal blood flow; total renal blood flow estimated as left kidney renal blood flow × 2.

* Represents effect of group

* *P *≤* *0.05

** *P *<* *0.01

*** *P *<* *0.001

# Represents effect of treatment with 7NI

# *P *≤* *0.05

## *P *<* *0.01

### *P *<* *0.001

† Represents an interaction between group and treatment

† *P *≤* *0.05

†† *P *<* *0.01

††† *P *<* *0.001.

#### Baseline renal function

Renal function parameters are listed in Table2. GFR was considerably higher in STNx fetuses, (approximately 50% higher). This higher GFR in the STNx fetuses was also evident when the subgroup of animals which had undergone measurement of RBF was examined separately. For this subgroup, GFR/kg of body weight was 0.93 ± 0.11 (*n* = 5) mL/min/kg in the untreated animals and 1.42 ± 0.16 (*n* = 8) mL/min/kg in the STNx group (*P* = 0.05). The corresponding values for GFR/g kidney weight were 0.18 ± 0.02 and 0.25 ± 0.02 mL/min/g (*P* = 0.06). While GFR was higher in the STNx fetuses, neither RBF nor FF were significantly elevated in the STNx group, possibly because of variability between animals as to whether RBF or FF or a combination of both factors accounted for the elevation in GFR.

**Table 2:** Fetal renal function during control and 7NI periods

	Control		7NI		*P* value
	Untreated	STNx	Untreated	STNx	
Urine flow rate (mL/min)	0.25 ± 0.04	0.45 ± 0.09	0.26 ± 0.04	0.46 ± 0.07	*
Urinary osmolality (mosm/kg H_2_O)	365 ± 12	339 ± 16	365 ± 17	325 ± 16	–
Free water clearance (mL/min)	−0.053 ± 0.01	−0.036 ± 0.02	−0.046 ± 0.01	−0.042 ± 0.02	–
Excretion rates					
Sodium (μmol/min/kg)	3.4 ± 0.8	7.6 ± 2.0	3.9 ± 0.7	8.6 ± 1.8	*
Potassium (μmol/min/kg)	1.5 ± 0.2	1.5 ± 0.2	1.3 ± 0.2	1.4 ± 0.2	#
Osmoles (μosm/min/kg)	18.0 ± 2.6	29.8 ± 5.3	18.0 ± 2.4	31.0 ± 4.6	*
Urinary Na/K	2.28 ± 0.44	4.75 ± 1.21	3.41 ± 0.50	6.48 ± 1.26	###
GFR (mL/min/g KW)	0.19 ± 0.02	0.28 ± 0.02	0.15 ± 0.02	0.25 ± 0.02	***,#
GFR (mL/min/kg BWT)	0.96 ± 0.11	1.50 ± 0.14	0.78 ± 0.07	1.34 ± 0.10	***,#
Filtration fraction (%) (GFR/RBF) × 100	5.9 ± 0.8	7.8 ± 1.0	5.5 ± 0.8	7.8 ± 1.0	–
Filtration fraction (%) (GFR/RPF) × 100	10.2 ± 1.6	13.2 ± 1.7	9.5 ± 1.3	13.2 ± 1.7	–
Tubular handling (Na, K)					
Filt_Na_ (μmol/min)	594 ± 91	974 ± 95	470 ± 70	888 ± 73	**,#
R_Na_ (μmol/min)	578 ± 89	938 ± 94	454 ± 68	847 ± 73	**,#
FR_Na_ (%)	97.0 ± 0.8	96.0 ± 1.0	96.4 ± 0.7	95.1 ± 1.0	–
R_Na_P (μmol/min)	381 ± 91	736 ± 98	281 ± 76	671 ± 85	**
FR_Na_P (%)	61.8 ± 6.1	73.0 ± 3.3	53.0 ± 8.0	71.3 ± 4.2	*
R_Na_D (μmol/min)	174 ± 25	224 ± 28	177 ± 28	211 ± 30	–
FR_Na_D (%)	35.0 ± 5.5	23.2 ± 2.3	43.1 ± 7.7	23.9 ± 3.1	*
DR_Na_DD (%)	92.4 ± 1.6	88.6 ± 2.4	90.5 ± 1.6	84.4 ± 2.5	–
Filt_K_ (μmol/min)	18.2 ± 2.8	28.3 ± 2.7	14.6 ± 2.2	25.7 ± 2.2	**,#
R_K_ (μmol/min)	10.3 ± 2.7	20.8 ± 2.5	8.3 ± 1.9	18.9 ± 2.0	**
FR_K_ (%)	49.4 ± 7.2	72.0 ± 2.7	53.4 ± 6.2	72.7 ± 3.2	**

Untreated *n* = 14 (except for tubular handling data where *n* = 10–11); STNx *n* = 16 (except for tubular handling data where *n* = 13–16). Filtration fraction values: Untreated *n* = 5, STNx *n* = 8.

U_Na_/K; urinary sodium to potassium ratio. KW, kidney weight; BWT, body weight; GFR, glomerular filtration rate; Filt_x_, filtered load of x; R_x_, FR_x_, absolute and fractional reabsorption of x; R_Na_P and R_Na_D, reabsorption of sodium by the proximal and distal tubules respectively; FR_Na_P and FR_Na_D, fractional reabsorption of sodium by the proximal and distal tubules respectively; DR_Na_DD, distal reabsorption of sodium as a % of distal delivery of sodium.

* Represents effect of group

* *P *≤* *0.05

** *P *<* *0.01

*** *P *<* *0.00

# Represents effect of treatment with 7NI

# *P *≤* *0.05

## *P *<* *0.01

### *P *<* *0.001

† Represents an interaction between group and treatment

† *P *≤* *0.05

†† *P *<* *0.01

††† *P *<* *0.001.

The higher GFR in the STNx fetuses was accompanied by an approximate doubling of urine flow rate and sodium and osmolar excretion rates in the STNx group. Due to the increased GFR, the amounts of filtered sodium, total reabsorbed sodium and sodium reabsorbed in the proximal tubule were higher in fetuses of STNx ewes, as were filtered potassium and reabsorbed potassium. Although there was no significant difference in overall fractional reabsorption of sodium, the fractional reabsorption of sodium in the proximal tubules was higher and the fractional reabsorption of sodium in the distal tubules was lower in fetuses of STNx ewes. Fractional reabsorption of potassium was also higher (Table2).

#### Baseline tubuloglomerular feedback responses

**Figure 1:**
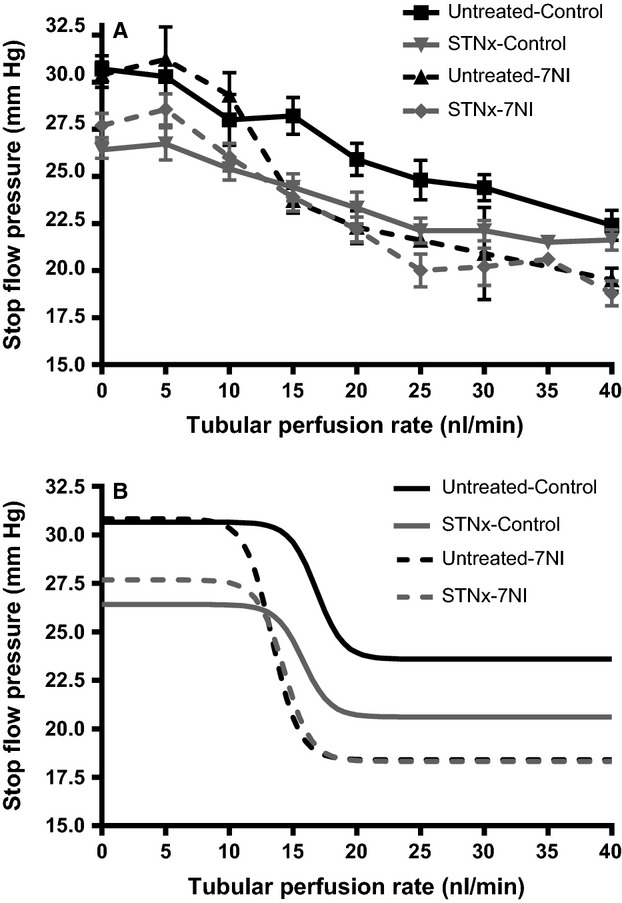
TGF curves during control and 7NI periods. (A) Raw data. (B) Stylized curves – The normalized data were fitted by means of a nonlinear least-squares curve fitting program (Selen et al. 1983; Brown et al. 2004).

While *P*_FF_ was no different, *P*_SF_ for STNx fetuses was significantly lower than untreated fetuses (Table3). As a consequence, the net filtration pressure for STNx fetuses was lower than that of untreated fetuses and the calculated filtration coefficient (*K*_f_) was three times higher (Table3). STNx fetuses had a moderately lower Δ*P*_SF_, but there was no difference in either %Δ*P*_SF_ or the turning point of the TGF curve between untreated and STNx fetuses (Fig.1, Table3).

**Table 3:** Micropuncture results for Untreated and STNx fetuses

	Control				7NI				*P* value
	*n*	Untreated	*n*	STNx	*n*	Untreated	*n*	STNx	
Free flow pressure (*P*_FF_) (mmHg)	24/7	7.2 ± 0.4	22/7	7.8 ± 0.3	15/3	8.0 ± 0.4	11/5	7.6 ± 0.4	–
Stop flow pressure (*P*_SF_) (mmHg)	10/5	30.9 ± 0.7	10/7	26.4 ± 0.4	13/4	31.3 ± 0.4	12/6	27.7 ± 0.5	***
Net filtration pressure (afferent end) (NFP_aff_) (mmHg)	5	24.4 ± 1.2	7	19.2 ± 0.4	4	25.2 ± 2.5	6	21.6 ± 1.7	**
Net filtration pressure (efferent end) (NFP_eff_) (mmHg)	4	24.2 ± 1.2	5	17.9 ± 0.5	4	24.3 ± 2.5	3	22.4 ± 2.9	*
Filtration coefficient (*K*_f_) (μL/min/g kidney/mmHg)	4	5.2 ± 0.2	5	19.0 ± 0.8	3	5.0 ± 1.1	3	14.6 ± 2.2	***
Change in stop flow pressure (Δ*P*_SF_) (mmHg)	8/5	7.2 ± 0.5	6/5	5.8 ± 0.2	6/4	12.4 ± 1.0	8/5	9.4 ± 1.1	*,###
%Δ*P*_SF_	7/5	22.5 ± 1.0	5/5	21.5 ± 0.7	5/4	38.8 ± 3.2	8/5	33.3 ± 3.4	###
Turning point (TP) (nL/min)	6/4	17.0 ± 1.0	7/6	15.0 ± 1.4	6/4	12.7 ± 0.8	8/5	13.6 ± 0.7	#

When n values are expressed as *x*/*y*, *x* is the number of tubules measured and *y* is the number of fetuses.

* Represents effect of group ^*^P ≤* *0.05

** *P *<* *0.01

*** *P *<* *0.001

# Represents effect of treatment with 7NI ^#^*P *≤* *0.05

## *P *<* *0.01

### *P *<* *0.001

† Represents an interaction between group and treatment ^†^*P *≤* *0.05

†† *P *<* *0.01

††† *P *<* *0.001.

### Effects of nNOS inhibition on cardiovascular function and blood composition

Untreated and STNx fetuses exhibited a number of similar changes in blood composition following inhibition of nNOS with 7NI. Fetal arterial Po_2_ fell while arterial Pco_2_ rose and a mild lactic acidemia developed (Table1). There were also small rises in hematocrit and plasma sodium level. However, there was an interaction between group and treatment for plasma potassium, such that there was a small increase in plasma potassium level following 7NI treatment in the untreated group, but no change in the STNX group (Table1). There was also an interaction between group and treatment for mean arterial pressure, such that mean arterial pressure fell in the untreated group, but was unchanged in the STNx group (Table1). By contrast, only treatment effects were evident in heart rate, abdominal aortic (AA) blood flow and RBF, all of which fell following 7NI (Table1). Because both abdominal aortic blood flow and RBF fell, when RBF was expressed as a percentage of AA blood flow there was no change in either group (Table1). Renal vascular resistance tended to increase after 7NI but this did not reach significance (Table1).

#### Effects of 7NI on renal function

Urine flow rate did not change after 7NI and the twofold higher rate for STNx fetuses observed in the control period remained evident (Table2). By contrast, there was a reduction in GFR after 7NI, and because of this there were corresponding falls in the filtered loads of sodium and potassium and total reabsorption of sodium. The urinary sodium to potassium ratio increased after 7NI and there was a small fall in potassium excretion (Table2). There were no interactions between group and treatment in any of these variables (Table2).

#### Effect of 7NI on the TGF response

After 7NI treatment, there were no changes in free flow pressure, stop flow pressure, net filtration pressure, or calculated *K*_f_ (Table3). By contrast, there were increases in *ΔP*_SF_ and %*ΔP*_SF_ and a reduction in the turning point of the TGF curve. No statistically significant interaction was observed between group and treatment (Fig.1, Table3). However, because of the small number of animals with available data for some variables this finding should be viewed with caution. In particular, there was a tendency for calculated *K*_f_ to be lower following 7NI in the STNx group (*P *=* *0.068 by unpaired *t* test).

The L-NAME treatment resulted in similar changes to those observed for 7NI treatment (Table4).

**Table 4:** Comparison of micropuncture results for control, L-NAME and systemic 7NI

	Control	*n*	Intratubular L-NAME	*n*	Systemic 7NI	*n*
Untreated						
*P*_FF_ (mmHg)	7.2 ± 0.4	24/7			8.0 ± 0.4	15/3
*P*_SF_ (mmHg)	30.9 ± 0.7	10/5	31.9 ± 0.5	7/4	31.3 ± 0.4	13/4
Δ*P*_SF_ (mmHg)	7.2 ± 0.5	8/5	10.7 ± 0.4**	7/4	12.4 ± 1.0**	6/4
%Δ*P*_SF_	22.5 ± 1.0	7/5	33.5 ± 1.1**	7/4	38.8 ± 3.2**	5/4
TP (nL/min)	17.0 ± 1.0	6/4	12.6 ± 0.7**	7/4	12.7 ± 0.8**	6/4
STNx						
*P*_FF_ (mmHg)	7.8 ± 0.3	22/7			7.6 ± 0.4	11/5
*P*_SF_ (mmHg)	26.4 ± 0.4	10/7	26.4 ± 0.9	8/4	27.7 ± 0.5	12/6
Δ*P*_SF_ (mmHg)	5.8 ± 0.2	6/5	7.5 ± 0.5	7/4	9.4 ± 1.1*	8/5
%Δ*P*_SF_	21.5 ± 0.7	5/5	28.1 ± 1.8	7/4	33.3 ± 3.4*	8/5
TP (nL/min)	15.0 ± 1.4	7/6	12.9 ± 1.2	7/4	13.6 ± 0.7	8/5

*n* values expressed as *x*/*y* with *x* being the number of tubules measured and *y* being the number of animals.

* *P* < 0.05

** *P* < 0.01, L-NAME or 7NI compared to Control; As the intratubular infusion of L-NAME occurred directly after control periods and before 7NI infusion, the control values for *P*_FF_ were used.

## Discussion

Previous studies from our group have reported that fetuses of STNx ewes are volume expanded, possibly due to an impaired ability of the maternal kidneys to maintain appropriate transplacental fluid flux and thus fetal fluid and electrolyte balance (Gibson et al. 2007). This state of volume expansion in fetal life was found to be resolved by 1–2 weeks after birth and hypothesized to be due to removal from the adverse intrauterine environment (Brandon et al. 2008, 2011). Interestingly, the current study shows that fetuses of STNx ewes which are only 10–15 days older than the previous studies (∼139 days vs. ∼127 days), did not exhibit signs of volume expansion, nor did the TGF curves resemble that of volume expanded fetuses that we have reported previously (Brown et al. 2011). This indicates that in the 10–15 day interim, the fetal kidney may have matured functionally and thus could deal with the increased fluid electrolyte flux and maintain its own homeostasis.

The older fetuses of STNx ewes did not appear to be volume expanded, that is, although their plasma chloride levels were slightly lower than those of the control group, the difference (1 mmol/L) was considerably less than we have found in younger fetuses (4 mmol/L). Their hemotcrits were not reduced and if anything, tended to be elevated compared to the controls and their plasma sodium levels were significantly elevated. However, they did exhibit alterations in renal function compared to fetuses of untreated ewes, namely an increase in GFR, urine flow rate and sodium excretion (Table2). Due to the increase in GFR, there was a doubling in the amount of sodium and potassium being filtered by the kidney. However, unlike in the previous report (Gibson et al. 2007) and consistent with a lack of volume expansion, the fractional reabsorption of sodium was not different in fetuses of STNx ewes compared to fetuses of untreated ewes, and the fractional reabsorption of sodium by the proximal tubule was increased rather than decreased. Thus, despite the hyperfiltration and consequent high filtered sodium load, proximal tubular reabsorption was maintained in STNx fetuses and glomerulotubular balance was achieved without the need for compensation by the distal tubule.

The hyperfiltration in fetuses of STNx ewes in the current study appears to be largely due to a very much higher *K*_f_. Net filtration pressure was lower in this group and mean arterial pressure and renal blood flow were not different to untreated fetuses. There was also no difference in free flow pressure (*P*_FF_) between fetuses of untreated and STNx ewes. Together these findings suggest that there is a larger surface area for filtration in fetuses of STNx ewes, along with an enlarged proximal tubule. *K*_f_ is a function of filtration surface area and the hydraulic conductivity of the filtration barrier, and although we cannot comment on differences in hydraulic conductivity we have previously reported that 1–2-week-old lambs born to STNx ewes had enlarged glomeruli (glomerular volume was increased by 30% while glomerular number was not altered) (Brandon et al. 2008). Since the fetuses in the current study were close to term, and it is recognized that nephron formation is complete by 130 days in the fetal sheep (Robillard et al. 1981), it is likely that the glomerular hypertrophy we observed in after birth in STNx offspring, was already present. This glomerular hypertrophy would at least partly explain the higher *K*_f_ and GFR of the STNx group. Evidence of glomerular hypertrophy as well as hypertrophy of proximal tubule cells has been found in adult rats after uninephrectomy (Pollock et al. 1992) and in fetal rats after maternal bilateral ureteral ligation (Okada and Morikawa 1990). The authors concluded that in these fetal rats, the proximal tubule enlargement was stimulated by increases in glomerular function due to maternal renal failure as a result of ureteral ligation (Okada and Morikawa 1990). Further investigations by the same group found that maternal uninephrectomy stimulated an increase in glomerular volume and morphological development of the proximal tubule in the fetal rat kidney (Okada et al. 1996).

Our finding of higher *K*_f_ being an important contributor to the higher GFR in fetuses of STNx ewes is consistent with other studies in developing animals. For example, a 7.5-fold increase in *K*_f_ was observed between 1 and 6 weeks in puppies after birth (Goldsmith et al. 1986), and increases in *K*_f_ contribute to the increase in GFR observed between birth and adulthood in both guinea pigs (Spitzer and Edelmann 1971) and Munich–Wistar rats (Ichikawa et al. 1979). Similarly in our own studies in sheep we found that *K*_f_ was higher in 12–18-day-old lambs than in late gestation fetuses (Turner et al. 2008).

Our values for filtration fraction (GFR/RPF) in this study (Untreated 10.2 ± 1.6%; STNx 13.2 ± 1.7%) were similar to those we have determined previously in chronically catheterized fetal sheep aged >125 days (12 ± 1%, (Hill and Lumbers 1988); 11.8 ± 1.8%, (Stevenson et al. 1996)) using different methodology (GFR measured as clearance of ^125^I sodium iothalamate, and RPF determined using the hematocrit and RBF measured with radioactive microspheres). The ratio of filtration fraction in the fetus compared to the adult sheep is 0.8 (Lumbers 2000).

Since NO has been shown to be an important regulator of TGF function, especially in response to volume expansion (Brown et al. 2004), the effects of inhibiting macula densa-derived nNOS were investigated in our model of mild maternal renal insufficiency. Inhibition of NO production by 7NI treatment resulted in a significant increase in reactivity (Δ*P*_SF_ and % Δ*P*_SF_ increased) in both untreated and STNx fetuses, as well as an increase in sensitivity (reduced TP) as expected from earlier work (Thorup and Persson 1996a).

To avoid any possible systemic effects of a NOS inhibitor, which may make interpretation of results difficult, it would be ideal to infuse the NOS inhibitor directly into the tubule. Thorup et al. (1993) infused L-NNA (a nonspecific NOS inhibitor) directly into the tubule and found that there was a progressive dose-dependent increase in TGF sensitivity, that is, the TGF curve shifted leftward and GFR fell. So it is clear that under normal conditions in adult animals TGF sensitivity is under the influence of NO production which makes the TGF system less responsive (Wilcox et al. 1992; Thorup and Persson 1994). 7NI is a specific nNOS inhibitor but the difficulty with its use is that it has a very low solubility in water and therefore cannot be administered directly into the tubule. When dissolved in peanut oil and administered i.p., it has been shown to have the same inhibitory effect as L-NNA or L-NAME (Ollerstam et al. 1997). Therefore, to confirm that the effects of NO inhibition on the TGF response that we have observed in the current study were specific to intrarenal NO, we examined the effects of an intratubular infusion of L-NAME on the TGF in a small number of animals. This meant that the possibly complicating effects of systemic administration of the nNOS inhibitor were avoided and the resulting differences in *ΔP*_SF_ and turning point were confirmed to be the result of inhibition of NOS production in the macula densa cells. The results in this sheep model clearly show that the i.p. 7NI has a similar effect on the TGF curve as a general NOS inhibitor administered via the tubular perfusate (Table4). Thus, we can conclude that inhibition of endogenous NO from the macula densa modulates the TGF response in the fetus in a similar manner to the adult, that is, the sensitivity increased (the curve shifted leftward) and reactivity increased (Δ*P*_SF_). These changes to the TGF curve appeared to be similar in both groups of fetuses and the *P* value for the interaction term of the ANOVA was >0.1 suggesting that a difference between the groups was unlikely. However, if power was increased by studying more animals, the tendency for *K*_f_ to fall in STNx fetuses following treatment with 7NI could be either confirmed or refuted.

In conclusion, although we hypothesized that the TGF curve of fetuses from STNx ewes would resemble that of a volume expanded fetus and they would respond differently to NO inhibition, this was not the case and likely reflects their normovolemic state. However, we did find that the stop flow pressure and net filtration pressure were greatly attenuated and *K*_f_ was much higher in the fetuses of STNx mothers, all indicating that glomerular hypertrophy is likely to be present in late gestation in utero, as it is after birth, in this model.

It is worth remembering that this model of mild maternal renal insufficiency results in ewes that are capable of becoming pregnant and maintaining normal fetal growth and development. Importantly, the differences we have observed in fetal renal function were present even though maternal renal function was only slightly impaired and would not necessarily be clinically apparent. Since hyperfiltration and glomerular hypertrophy are important factors in the pathophysiology of renal disease and hypertension (Brenner et al. 1996), it is reasonable to speculate that offspring of mothers with subclinical renal dysfunction may develop hypertension and/or renal dysfunction later in life.
